# Memantine Inhibits α3β2-nAChRs-Mediated Nitrergic Neurogenic Vasodilation in Porcine Basilar Arteries

**DOI:** 10.1371/journal.pone.0040326

**Published:** 2012-07-05

**Authors:** Reggie Hui-Chao Lee, Ting-Yi Tseng, Celeste Yin-Chieh Wu, Po-Yi Chen, Mei-Fang Chen, Jon-Son Kuo, Tony Jer-Fu Lee

**Affiliations:** 1 Institute of Medical Sciences, College of Medicine, Tzu Chi University, Hualien, Taiwan; 2 Institute of Pharmacology & Toxicology, College of Medicine, Tzu Chi University, Hualien, Taiwan; 3 Institute of Life Sciences, College of Life Sciences, Tzu Chi University, Hualien, Taiwan; 4 Center for Vascular Medicine, College of Life Sciences, Tzu Chi University, Hualien, Taiwan; 5 Department of Research, Buddhist Tzu Chi General Hospital, Hualien, Taiwan; 6 Department of Pharmacology, Southern Illinois University School of Medicine, Springfield, Illinois, United States of America; Kaohsiung Chang Gung Memorial Hospital, Taiwan

## Abstract

Memantine, an NMDA receptor antagonist used for treatment of Alzheimer’s disease (AD), is known to block the nicotinic acetylcholine receptors (nAChRs) in the central nervous system (CNS). In the present study, we examined by wire myography if memantine inhibited α3β2-nAChRs located on cerebral perivascular sympathetic nerve terminals originating in the superior cervical ganglion (SCG), thus, leading to inhibition of nicotine-induced nitrergic neurogenic dilation of isolated porcine basilar arteries. Memantine concentration-dependently blocked nicotine-induced neurogenic dilation of endothelium-denuded basilar arteries without affecting that induced by transmural nerve stimulation, sodium nitroprusside, or isoproterenol. Furthermore, memantine significantly inhibited nicotine-elicited inward currents in Xenopous oocytes expressing α3β2-, α7**-** or α4β2**-**nAChR, and nicotine-induced calcium influx in cultured rat SCG neurons. These results suggest that memantine is a non-specific antagonist for nAChR. By directly inhibiting α3β2-nAChRs located on the sympathetic nerve terminals, memantine blocks nicotine-induced neurogenic vasodilation of the porcine basilar arteries. This effect of memantine is expected to reduce the blood supply to the brain stem and possibly other brain regions, thus, decreasing its clinical efficacy in the treatment of Alzheimer’s disease.

## Introduction

Alzheimer’s disease (AD) is a progressive neurodegeneration disease. The beta-amyloid peptide (Aβ) and the hyperphosphorylated microtubular protein tau are the key causative factors in pathogenesis of AD [1]. The oxidation of Aβ and hyperphosphorylated protein tau further triggers lipid peroxidation and inflammation, leading to irreversible loss of neurons [2], particularly in the hippocampus and cortex in AD. The loss of neurons results in impairments in learning, memory, decision making, language, and orientation to physical surroundings [3].

In AD patients, excessive accumulation of extracellular Aβ in the brain inhibits the function of glutamate transporter in astrocytes, leading to glutamate excitotoxicity in neurons [4]. Memantine, an N-methyl-D-aspartate receptor (NMDA) antagonist [5], [6], via inhibition of the NMDA receptor on neurons, prevents neuronal cell death triggered by excessive extracellular calcium influx induced by NMDA over stimulation, thus improving the cognitive impairments in AD patients [4], [6], [7]. The neuroprotective effect of memantine has been shown to reduce the perfusion-reperfusion-induced neuronal damage [8], [9]. Memantine, a novel drug with less clinical adverse events nowadays, has been approved for treating moderate to severe AD by the Food and Drug Administration [4], [5], [10], [11]. However, memantine has been shown to inhibit α4β2-, α9α10-, and α7-nicotinic acetylcholine receptors (nAChRs) in the central nervous system (CNS) [12]–[15]. Meanwhile, inhibition of α7-nAChRs [16], [17] and α3β2-nAChRs [18] located on cerebral perivascular sympathetic nerves will result in decreased dilation (i.e., constriction) of the basilar artery. The expected diminished blood flow, at least, in the brain stem region induced by memantine may aggravate the already diminished cerebral blood flow in AD patients [19], [20]. It has been reported that the vertebrobasilar insufficiency is a cause of dementia and progressive loss of memory [21], [22]. Therefore, inhibition of nAChR-mediated basilar arterial neurogenic vasodilation by memantine may become an important side effect of its clinical sue. Accordingly, effects of memantine on the nAChR-mediated neurogenic nitrergic vasodilation in the basilar artery were examined. Our results indicated that memantine inhibited nicotine-induced α3β2-nAChR-mediated neurogenic nitrergic dilation of isolated basilar arteries.

## Results

### Effects of Memantine on Transmural Nerve Stimulation (TNS)- and Nicotine-induced Neurogenic Vasodilations

**Figure 1:**
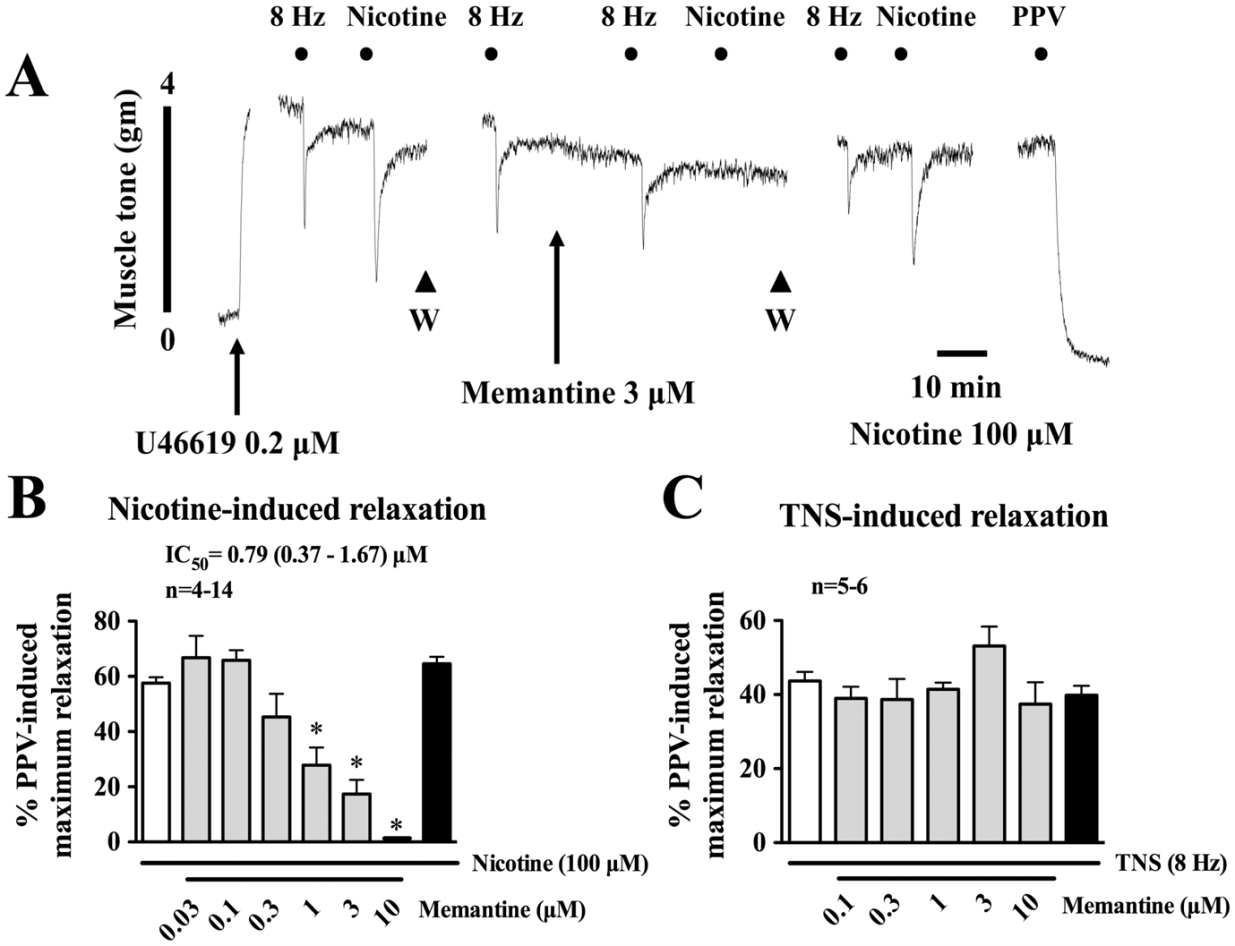
Effects of memantine on α3β2-nAChR-mediated nicotine-induced vasodilation in porcine basilar arteries. All experiments were carried out in endothelium-denuded porcine basilar arteries in the presence of active muscle tone induced by U46619 (0.2 µM). A representative tracing showing that memantine (3 µM) blocked nicotine (100 µM)-induced vasodilation (panel A) without affecting that elicited by TNS (8 Hz). Summaries of memantine blockade of nicotine (100 µM)-induced vasodilation with IC_50_ values are shown in panel B. Failure of memantine to inhibit relaxation elicited by TNS is summarized in panel C. Arrowheads in panel A indicate repeated washings (W). Vasodilation is estimated as percent of papaverine (PPV, 100 µM)-induced maximum vasodilation. Values are means ± SEM; n indicates number of experiments. *P<0.05 indicates significantly different from control.

Our previous studies have demonstrated that TNS at 8 Hz and nicotine at 100 µM induce maximum neurogenic vasodilation in porcine isolated basilar arteries [16], [18]. These parameters, therefore, were used in the present studies. In the presence of active muscle tone induced by a thromboxane A2 analog, 9,11-Dideoxy-11α,9α-epoxymethanoprostaglandin F_2α_ (U46619), endothelium-denuded basilar arterial rings relaxed upon application of TNS (8 Hz) or nicotine (100 µM) (Figure 1A). The nicotine-induced relaxation was inhibited by memantine (Figure 1A and 1B) in a concentration-dependent manner. The IC_50_ value for memantine in inhibiting nicotine-induced relaxation was 0.79 (0.37–1.67) µM. Nicotine-induced relaxation was fully recovered after washing off memantine (Figure 1A and 1B). In contrast, memantine did not significantly affect the TNS-induced vasorelaxation (Figure 1A and 1C), suggesting that inhibition of nicotine-induced vasorelaxation by memantine is not due to possible local anesthetic or nonspecific effects.

### Effects of Memantine on Isoproterenol (ISO)- or Sodium Nitroprusside (SNP)-induced Vasodilation

**Figure 2:**
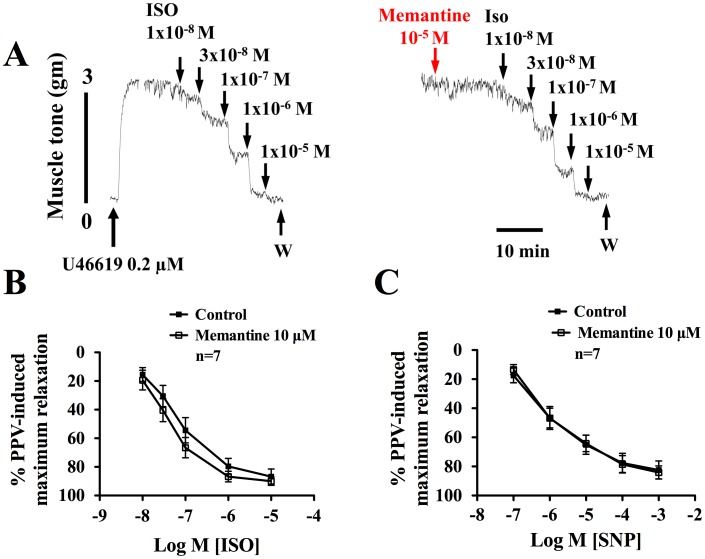
Effects of memantine on isoproterenol (ISO)- and sodium nitroprusside (SNP)-induced vasorelaxation. All experiments were carried out in endothelium-denuded porcine basilar arteries in the presence of active muscle tone induced by U46619 (0.2 µM). Panel A, a representative tracing showing that ISO (10^−8^ M–10^−5^ M) induced a concentration-dependent vasodilation which was not affected by memantine (10 µM). In panels B and C, concentration-response curves showing effects of memantine on ISO- and SNP-induced relaxation in porcine basilar arteries. The EC_50_ values of ISO in inducing relaxation in the presence and absence of memantine were 0.06 (0.02–0.18) µM and 0.03 (0.01–0.09) µM, respectively. The EC_50_ values of SNP in inducing relaxation in the presence and absence of memantine were 0.61 (0.2–2) µM and 0.71 (0.29–1.76) µM, respectively. The EC_50_ values for isoproterenol and sodium nitroprusside-induced relaxation were not significantly different between the control and that in the presence of memantine (*p*>0.05). The values are mean ± SEM. n, number of experiments. Relaxation is estimated as percent of PPV (100 µM)-induced maximum relaxation.

In the presence of active muscle tone induced by U46619, SNP (0.1 µM to 1 mM) (Figure 2C) and ISO (10 nM to 10 µM) (Figures 2A and 2B) in concentration-dependent manner relaxed endothelium-denuded porcine basilar arteries. Memantine at 10 µM did not affect the concentration-dependent vasodilation curves induced by ISO (Figure 2B) or SNP (Figure 2C). The EC_50_ values for ISO in control and in the presence of memantine were 0.06 (0.02–0.18) µM and 0.03 (0.01–0.09) µM (*p*>0.05), respectively, and those for SNP were 0.61 (0.2–2) µM and 0.71 (0.29–1.76) µM (*p*>0.05), respectively.

### Memantine Inhibited α7-, α4β2-, and α3β2-nAChR-mediated Inward Currents

**Figure 3:**
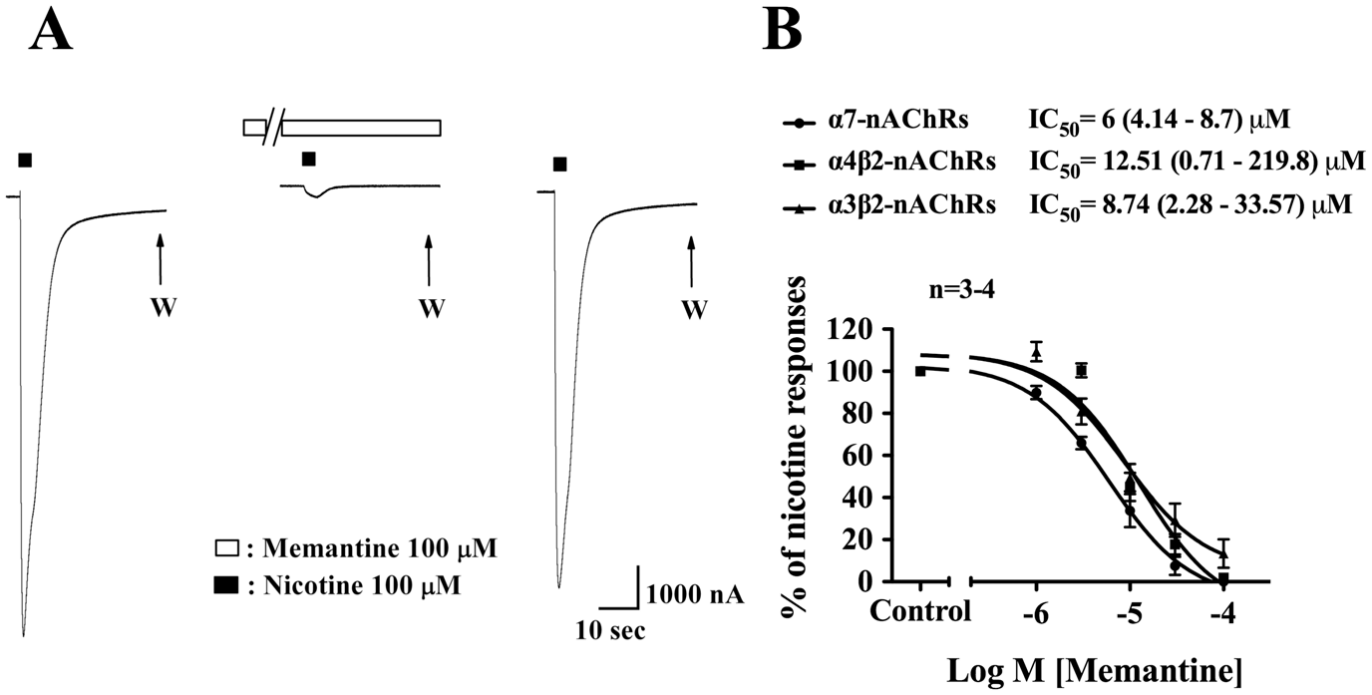
Effects of memantine on nicotine-induced inward currents in *xenopus* oocytes expressing α7-nAChRs, α4β2-nAChRs, or α3β2-nAChRs. A representative tracing in panel A from two-electrode voltage clamp studies shows that memantine (100 µM) blocks the inward currents elicited by nicotine (100 µM) in an oocyte expressing α4β2-nAChR. Oocytes were continuously perfused with memantine for 5 mins as indicated by the long horizontal open bar. Short horizontal solid bar above the tracing in panel A denotes application of nicotine for 3 sec. The blockade was completely reversed after washing off memantine. In panel B, memantine in a concentration-dependent manner blocked α7-, α4β2-, and α3β2-nAChRs-mediated inward currents elicited by nicotine (100 µM) with the IC_50_ values as indicated. Arrows in panel A indicate repeated washings (W). Inward currents were estimated as a percentage of those induced by 100 µM nicotine. Values are means ± SEM; n indicates number of experiments.

We have demonstrated that both α3β2-nAChR and α7-nAChR located on cerebral sympathetic neurons mediate cerebral nitrergic vasodilation [16], [18]. To investigate a possible direct effect of memantine on nAChRs, we used α7-, α4β2-, and α3β2-nAChR-overexpressing oocytes to determine if memantine affected the inward currents mediated by these different subtypes of nAChR. In two-electrode voltage clamp recording, nicotine-induced, α7-, α4β2-, and α3β2-nAChR-mediated inward currents in the oocytes were inhibited by memantine in a concentration-dependent manner (Figure 3A and 3B), and the inhibition was fully recovered after washing off memantine (Figure 3A). The IC_50_ values for memantine in inhibition of α7-, α4β2-, and α3β2-nAChR-mediated inward currents were 6.00 (4.14–8.70) µM, 12.51 (0.71–219.80) µM, and 8.74 (2.28–33.57) µM, respectively (Figure 3B).

### Effects of Memantine on Nicotine- and KCl-induced Calcium Influx in Cultured SCG Neurons

SCG neurons are the origin of cerebral perivascular sympathetic nerves. Our previous reports have indicated that cultured porcine SCG neurons contain dense nAChRs [23] which form membrane cation channels in controlling high calcium permeability [24]. Therefore, we used fluo-4, AM, an intracellular calcium imaging indicator, to determine whether calcium influx via activation of nAChRs in SCG neurons by nicotine would be inhibited by memantine. We have demonstrated that cultured SCG neurons from rats or pigs have similar pharmacological properties in responding to nAChR agonists and antagonists [25]. We chose to use cultured rat SCG neurons in this study due to significantly better cell viability of the cultured rat SCG neurons than that of the cultured porcine SCG neurons.

**Figure 4:**
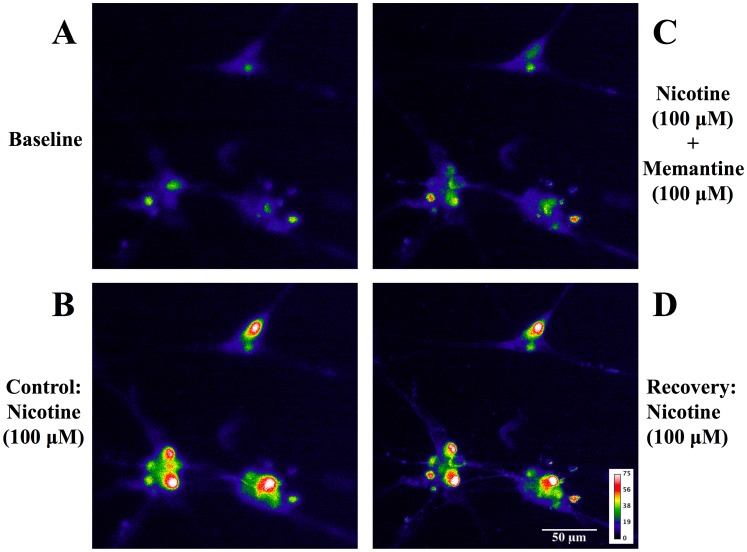
Effects of memantine on nicotine-induced calcium influx in cultured rat superior cervical ganglion (SCG) neurons. The neurons were loaded with fluo-4, AM (1 µM) in physiologic buffer and incubated at room temperature for 30 mins. Panel A showing the basal calcium image in each cell. In panel B, nicotine (100 µM) was applied to the medium to induce significant calcium influx into the neurons. Panel C showing that memantine (100 µM) significantly inhibited nicotine-induced calcium influx. In panel D, a complete recovery from the blockade of nicotine-induced calcium influx by memantine was observed 15 mins after washing off memantine. Values are means ± SEM; n indicates number of experiments. *P<0.05 indicates significantly different from control.

**Figure 5:**
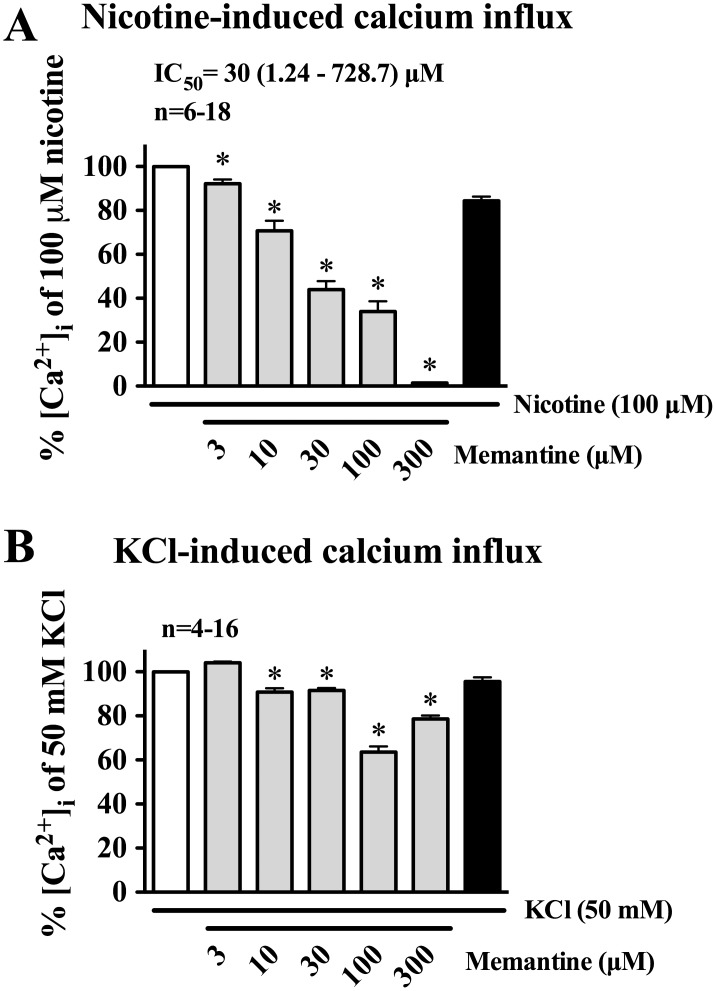
Summaries of effects of memantine on calcium influxes induced by nicotine (A) and KCl (B) in cultured neurons of the rat superior cervical ganglion (SCG). The SCG neurons were loaded with fluo-4, AM (1 µM) in physiologic buffer and incubated at room temperature for 30 mins. Nicotine (100 µM) or KCl (50 mM) was then applied and the intracellular calcium measured as shown in Figure 4. Memantine in 3 µM to 300 µM was added 15 mins before application of nicotine (100 µM, panel A) or KCl (50 mM, panel B) to induce calcium influx, [Ca^2+^]_i_. Each concentration of memantine was examined separately in each preparation. Memantine concentration-dependently inhibited nicotine (100 µM)-induced calcium influx (with IC_50_ values shown in panel A). Effects of memantine on KCl (50 mM)-induced calcium influx are summarized and shown in panel B. Changes in intracellular calcium is estimated as percent of nicotine (100 µM)- or KCl (50 mM)-induced calcium influx. Values are means ± SEM; n indicates number of experiments. *P<0.05 indicates significantly different from the respective control.

Photographs in Figure 4 show a typical experiment by intracellular calcium imaging in the cultured SCG neurons, demonstrating that nicotine significantly increased calcium influx (Figures 4B and 5A). The increase was markedly inhibited by 15 min pretreatment of mamentine (Figure 4C vs. Figure 4B) in concentration-dependent manner (Figure 5A), and the inhibition was fully recovered 15 min after washing off memantine (Figure 4D). During this recovery phase, nicotine (100 µM) significantly increased calcium influx in the SCG neurons (Figures 4D and 5A). The IC_50_ value for memantine against nAChR-mediated calcium influx was 30.00 (1.24–728.7) µM (Figure 5A).

Memantine at 3 µM did not inhibit KCl (50 mM)-induced calcium influx (Figure 5B). However, memantine at 10, 30, 100, and 300 µM slightly but significantly inhibited the KCl-induced calcium influx by 9.23±1.81%, 8.5±1.12, 36.45±2.61, and 21.43±1.6%, respectively (Figure 5B). The KCl-induced calcium influx was fully recovered 15 min after washing off memantine (Figure 5B).

## Discussion

The major findings of the present study are that 1) in the endothelium-denuded cerebral arterial rings of the pigs crossbreed among Landrace, Yorkshire and Duroc (LYD), memantine in a concentration-dependent manner inhibits nicotine-induced vasorelaxation without affecting that induced by TNS, ISO- or SNP, 2) in oocytes expressing α7-, α4β2-, and α3β2-nAChRs, memantine in a concentration-dependent manner inhibits nicotine-induced inward currents mediated by all subtypes, and 3) in cultured rat SCG neurons, memantine in a concentration-dependent manner inhibits nicotine-induced calcium influx. These results suggest that memantine directly inhibits nAChRs, and, specifically, the α3β2-nAChR located on the sympathetic nerve terminals innervating the basilar arteries of the LYD pigs, leading to blockade of nicotine-induced neurogenic nitrergic dilation of these arteries.

It has been shown that nicotinic agonist-induced neurogenic nitrergic dilation of basilar arteries is mediated by α7-nAChR located on cerebral perivascular sympathetic nerves in pigs crossbred between Landrace and Yorkshire (LY) in USA [16], and by α3β2-nAChRs in LYD pigs in Taiwan [18]. In the present study, the concentration-dependent inhibition by memantine of nicotine-induced dilation of basilar arteries of the LYD pigs suggests that memantine inhibited the α3β2-nAChRs. Memantine, however, did not affect dilation of the basilar arteries induced by TNS, suggesting that memantine inhibition is not due to possible local anesthetic or nonspecific effects.

**Figure 6:**
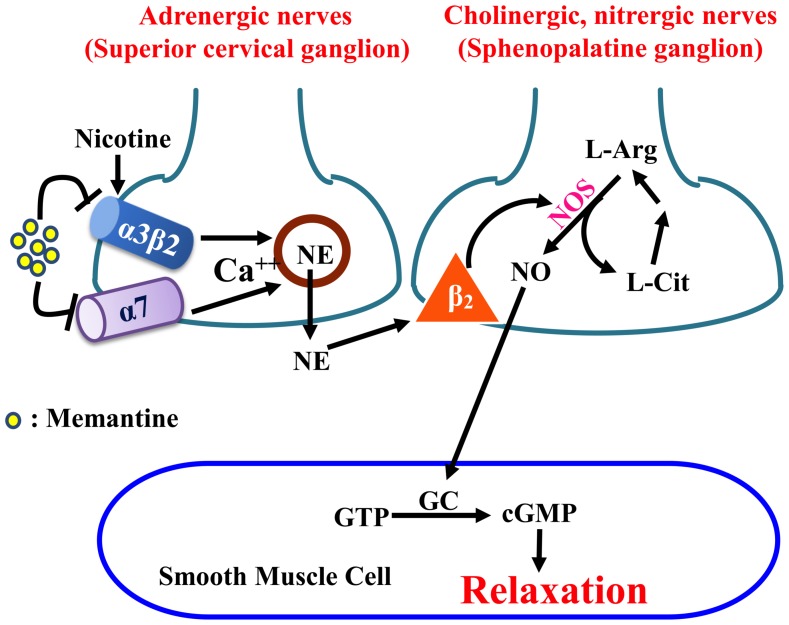
Schematic illustration of the role of memantine in modulating parasympathetic nitrergic dilation of basilar arteries. An axo-axonal interaction between adrenergic and nitrergic nerves in regulating vascular tone of the large cerebral arteries at the base of the porcine brain is shown. Nicotine activates presynaptic α3β2-nAChRs (in pigs crossbred among Landrace, Yorkshire and Duroc) or α7-nAChRs (in pigs crossbred between Landrace and Yorkshire) [16], [18] on sympathetic nerves causing release of NE which then activates the presynaptic β2-adrenoceptors located on neighboring nitrergic nerves, causing NO release from nitrergic nerve terminals. NO activates guanylate cyclase (GC) in the smooth muscle cell to increase the synthesis of cGMP from GTP and relaxes the smooth muscle. NO is synthesized from L-arginine (L-Arg) in the presence of NO synthase (NOS). The byproduct of NO synthesis, L-citrulline (L-Cit), is actively converted to L-Arg [49]. Memantine inhibits both presynaptic α7- and α3β2-nAChR-mediated nitrergic vasodilation.

According to our axo-axonal interaction hypothesis (Figure 6), the β2-adrenoceptor located on the perivascular nitrergic nerves plays an important role in regulating NE-induced nitric oxide (NO) release from the nitrergic nerve terminals, leading to vasodilation via the NO-cyclic guanosine monophosphate (cGMP) coupling [16], [18], [26], [27]. Therefore, activation of β-adrenoceptors by ISO or activation of cGMP coupling by SNP is expected to induce vasodilation of the basilar artery. The vasodilation induced by these vasodilators, however, was not affected by memantine. These results suggest that memantine inhibition of neurogenic vasodilation induced by nicotine was not due to blockade of presynaptic β2-adrenoceptors or postsynaptic β1-adrenoceptors [27], [28], or NO-cGMP coupling pathway [16]. This latter finding is further supported by lack of effect of memantine on nitrergic neurogenic vasodilation upon depolarization of perivascular nerves by TNS, suggesting that synthesis and release of NO or NO-cGMP coupling induced by electrical depolarization is not affected by memantine.

Memantine has been reported to inhibit α4β2-, α9α10-, and α7-nAChRs in the CNS [12]–[15], suggesting that memantine is non-specific for nAChR-subtypes. This is supported further by results of the present findings indicating that memantine inhibits nicotine-induced α3β2-, α7-, and α4β2-nAChR-mediated inward currents in oocytes and α3β2-mediated nitrergic vasodilation. These findings indicate that mamentine inhibits neurogenic vasodilation of basilar arteries primarily expressing the α3β2-nAChRs in the LYD pigs [18] or the α7-nAChR in the LY pigs [16], [18].

The perivascular sympathetic nerves to brain blood vessels originate in the SCGs [17], [29]. The rat SCG neurons contain α3β2- and α7-nAChRs [30]–[32]. The nAChRs in these neurons, like those in perivascular sympathetic nerves of porcine basilar arteries, mediate intracellular calcium influx [25]. We therefore determined effects of memantine on nAChRs in mediating calcium influx in cultured rat SCG neurons. Results from intracellular calcium image study demonstrated that memantine in a concentration-dependent manner inhibited nicotine-induced calcium influx (Fig. 5). These results further support the notion that α3β2- and/or α7-nAChRs are responsible for initiating calcium influx in nicotine-induced vasodilation of the basilar artery (Figure 6).

It was reported that activation of α3β2-nAChRs on human neuroblastoma SH-SY5Y cells induced a sustained elevation of intracellular Ca^2+^ levels which is highly dependent on the activation of voltage-operated calcium channels (VOCC) [33]. In the present study, memantine in concentrations of 10, 30, 100, and 300 µM inhibited KCl-induced calcium influx by 9.2±1.8%, 8.5±1.1, 36. 5±2.6, and 21.4±1.6%, respectively, while it exhibited greater inhibition on nicotine-induced calcium influx by 29.3±4.7, 56.1±3.9, 66.1±4.7, and 98.66±0.3%, respectively. These results further support a preferential inhibition by memantine on the nAChRs in addition to a nonspecific inhibition by memantine of the VOCC. Together with the findings in *Xenopus* oocytes (Figure 3), it is suggested that memantine inhibition of nicotine-induced inward currents or calcium influx is mainly via specific inhibition of α3β2- and/or α7-nAChRs. The exact mechanism of action, however, remains to be determined.

Functional nAChRs of α7- and α3β2-subtypes are present on cerebral perivascular sympathetic nerves [16], [18], which are activated by endogenous acetylcholine (ACh) and choline [16], [34], [35]. The significant concentrations of ACh and choline in cerebral spinal fluid [36], [37], therefore, would logically predict that sympathetic nAChRs are physiologically modulated in vivo, leading to nitrergic neurogenic vasodilation via the axo-axonal interaction mechanism (Figure 6). The decreased ACh release and the loss of cholinergic function in diseases such as the Alzheimer’s disease [38] are expected to exhibit less nitrergic vasodilation as that found in Alzheimer’s disease [39], [40]. Inhibition of sympathetic nAChRs by memantine as shown in the present studies would further aggravate the already diminished vasodilation. It has been shown that large cerebral arteries are important determinants of local microvascular pressure and also contribute significantly to total cerebral resistance [41]. Accordingly, change of blood flow by memantine in large artery like the basilar artery is an indication of decreased blood flow in the brain stem.

AD patients are treated with a recommended maintenance dose of memantine 30 mg/day, and its concentrations found in the serum and cerebrospinal fluid are 0.5 and 0.3 µM, respectively [42]. Furthermore, memantine concentrations as high as 1 µM may be reached in the extracellular brain compartment of patients receiving chronic 20 mg/day memantine [43]. Following 7 days of treatment of memantine (20 mg/day) in rats, the brain tissue concentrations of memantine are 25.9 µM which are 44-fold higher than the concentration of memantine in the serum [44]. These concentrations of memantine in extracellular brain compartment and brain tissue are expected to significantly inhibit nAChR-mediated neurogenic vasodilation (Figure 1), suggesting the possibility of worsening the already decreased regional cerebral blood flow in AD patients and accelerating the pathological progress [19], [20].

Furthermore, memantine inhibition of nAChR-mediated basilar arterial vasodilation suggests that the vertebrobasilar insufficiency may occur when AD patients are treated with memantine. The cerebrovascular insufficiency in posterior cerebral and vertebrobasilar systems which supply blood to posterior part of cerebral hemispheres (occipital lobe and temporal lobe), brainstem, and cerebellum, has been reported as a cause of transient global amnesia [21], [22]. The patients with transient global amnesia may suffer an amnesic stroke with permanent memory loss eventually [22]. Furthermore, vertebrobasilar insufficiency also causes dementia and progressive loss of memory via infarctions and ischemia in cortical and limbic systems and para-amygdaloid area [21]. Accordingly, our findings may offer a new explanation for the lack of efficacy of memantine in mild AD [45]. The optimal doses of memantine and its concentrations in the circulation and the cerebral spinal fluid for balancing its neurovascular effects may need to be monitored closely.

## Materials and Methods

### General Procedure

Fresh heads of adult pigs (90–110 kg) of either sex, crossbreed among Landrace, Yorkshire and Duroc (LYD), were collected at a local packing company (Hsien Meat Market Company Limited, Fong-lin county, Hualian, Taiwan). The entire brain, with dura mater attached, was removed and placed in Krebs’ solution equilibrated with 95% O_2_ balanced with 5% CO_2_ at room temperature. The composition of the Krebs’ solution is as follows: 122.0 mM NaCl, 5.16 mM KCl, 1.2 mM CaCl_2_, 1.22 mM MgSO_4_, 25.6 mM NaHCO_3_, 0.03 mM ethylenediamine-tetraacetic acid, 0.1 mM L-ascorbic acid, and 11.0 mM glucose with a final pH of 7.4. The basilar artery was dissected and cleaned of any connective tissue under a dissecting microscope, then processed for tissue bath myography study [26]. All protocols were approved by the Animal Experimentation Committee of the Tzu Chi University.

### Wire Myography

The cerebral arterial tension is mainly controlled by the perivascular nerve fibers, smooth muscle cells and endothelial cells. The net results of vasodilation and constriction induced by multiple mediators released from the perivascular nerves and endothelial cells determine the cerebral arterial tension. In the present study, we only focused on the effects of memantine on neurogenic control of cerebral arterial tone, i.e., neurovascular transmission. Therefore, possible influence by memantine on endothelial cells-mediated vasomotor activity was excluded by removing the endothelium. The endothelial denudation of all arterial ring segments was carried mechanically by a standard, brief, gentle rubbing of the intimal surface with a stainless-steel rod having a diameter (22–30 gauge) equivalent to the lumen of the arteries [26], [27]. An arterial ring segment (4 mm long) was cannulated with a stainless-steel rod (30-gauge hemispherical section) and a short piece of platinum wire, and mounted horizontally in a plastic tissue bath containing 9 ml Krebs’ solution maintained at 37°C. The platinum wire was bent into a U shape and anchored to a gate. The stainless-steel rod was connected to a strain-gauge transducer for isometric recording of changes in force [29]. The ring was equilibrated in the Krebs’ solution for 30 mins and then stretched to a resting tension of 750 mg [26], [27]. The ring segments were then contracted with U-46619 (0.3 to 3 µM) to induce an active muscle tone of 0.5–0.75 gm. Vasorelaxation was then induced by nicotine at 100 µM or transmural nerve stimulation (TNS) at 8 Hz in frequency, 0.6 ms in pulse duration and 200 mA in intensity for 25 s. The TNS-elicited relaxation served as a control for comparison with the nicotine-induced relaxation. After the TNS- and nicotine-induced relaxations were demonstrated, the arteries were washed with pre-warmed Krebs’ solution. A similar magnitude of active muscle tone was induced with U-46619 again. Memantine in different concentrations was administered 15 mins before repeating the TNS and application of nicotine, and one arterial ring preparation was used to examine effect of one concentration of memantine. IC_50_ values (the concentration that inhibits 50% of the maximum relaxation) of memantine on nicotine- or TNS-induced relaxation were determined. In order to avoid the possible development of tachyphylaxis on repeated applications of nicotine, at least 5 washes every 15 mins for totally 75 mins were performed before the next application of nicotine [16], [27]. At the end of each experiment, the complete removal of endothelial cells was verified by lack of changes in basal tone upon application of nitro-L-arginine (L-NNA), a NOS inhibitor; while papaverine (100 µM) was added to induce maximum relaxation. The magnitude of a vasodilator response was expressed as a percentage of the maximum response induced by papaverine [26].

For examining effects of memantine on relaxation induced by isoproterenol (ISO) or sodium nitroprusside (SNP), concentration-relaxation relationships for these two vasodilators were first obtained by cumulative concentrations in the endothelium-denuded arterial ring in the presence of active muscle tone induced by U-46619. After the rings were washed with prewarmed Krebs’ solution, a similar magnitude of active muscle tone was again induced by U-46619, and mamentine was then added. Fifteen mins later, concentration-relaxation response relationships for ISO or SNP were repeated. EC_50_ values (the concentration that induces 50% of the maximum relaxation) of ISO and SNP were determined. From these values, the geometric means of IC_50_ or EC_50_ values with 95% confidence intervals [46] were calculated.

### Two-electrode Voltage Clamp Recording

The expression of α3β2-, α4β2-, and α7-nAChRs on oocytes was carried out according to our previous reports [18], [34]. A modified two-electrode voltage clamp recording according to our previous report [34] was used. Two days after the injection of human α3, α4, α7, and β2 subunit cRNAs, membrane currents of the oocytes were recorded in a chamber consisting of 1.3 ml cylindrical well at room temperature. During the recording, the oocytes were continuously perfused with the bath solution at a rate of 10 ml/min.

Two-electrode voltage clamp for the whole oocyte recording was performed by an amplifier (model OC-725C, Warner Instruments, Hamden, CT, USA). The borosilicate glass capillaries (1.5 mm OD; World Precision Instruments, Sarasota, FL, USA) were pulled by a microelectrode puller (model P-97, Sutter, Novato, CA, USA). The resistance of an electrode filled with 3 M KCl was 0.2–1 MΩ. The membrane potential was held at -60 mV. Data acquisition and analysis were performed with pClamp 9.0 and Digidata 1322A (Axon Instruments, Union City, CA, USA). The input signals were filtered at 1 kHz and sampled at 2 kHz.

The magnitude of inward current induced by nicotine was determined by the current amplitude. To compensate for the difference in the nAChR expression level, the current amplitudes were normalized and expressed as percent of nicotine (100 µM)-induced response. Nicotine (100 µM) was applied for 3 sec at 15 mins interval. For examination of effects of memantine on nicotine-induced responses, memantine dissolved in the bath solution were perfused continuously into the bath chamber for 15 mins. Nicotine was then applied directly onto the oocytes to ensure its rapid interaction with α3β2-, α4β2-, or α7-nAChRs. IC_50_ values of memantine on nicotine-induced inward currents with 95% confidence intervals [46] were then calculated. After effects of memantine on nicotine-induced inward currents were established and the currents returned to the baseline, the memantine and nicotine were washed off with bath solution. 15 mins later, the response to nicotine alone was repeated to obtain an additional control. Between drug applications, the oocytes were perfused continuously with bath solution.

### Culture of the SCG Neurons

A modified method of culturing SCG neurons according to our previous reports [25], [47] was used in the present study. Primary SCG neuronal cultures were prepared from newborn Sprague-Dawley rat pups (2–8 days old) killed with sodium pentobarbital. Freshly dissected SCGs were placed in cold Hibernate A (Invitrogen) solution and cut into small pieces. Then, the ganglia were transferred to Mg^2+^/Ca^2+^-free Hanks’ balanced salt solution (HBSS) containing trypsin (2.5 mg/ml; Sigma-Aldrich) and were incubated for 30 mins at 37°C. Cells were released by gentle trituration with a fire-polished glass pipette at the end of the incubation. The cell suspension was centrifuged at 300 g for 5 mins. The pellet was gently resuspended in Neurobasal culture medium (Invitrogen) containing B27 (1∶50 dilution; Invitrogen), 0.5 mM L-glutamate (Gibco), 10 µM uridine (Sigma-Aldrich), 10 µM 5-Fluoro-5′ deoxyuridine (Sigma-Aldrich) and nerve growth factor (100 ng/ml; Invitrogen). All media and HBSS contained 100 units/ml penicillin and 100 units/ml streptomycin. The single-cell suspension was plated onto a 4-well culture plate with a rat-tail collagen (Gibco)-coated glass coverslip (12-mm diameter; Fisher Scientific) in each well and incubated with air containing 5% CO_2_ at 37°C. The growth medium was changed once on day 2. The SCG cells were stained with anti-rabbit neurofilament 200 (Sigma-Aldrich) as a marker of neuronal cells.

### Intracellular Calcium Imaging

A modified intracellular calcium image according to our previous report [48] was used. In culture for 3–7 days, the SCG cells were examined for effects of nicotine and KCl on calcium influx by confocal microscopy. The cells were washed with physiologic buffer (130 mM NaCl, 5 mM KCl, 10 mM HEPES, 5 mM glucose, 2 mM CaCl_2_, 2 mMMgCl_2_, pH 7.3), and were loaded with 1 µM fluo-4, AM in physiologic buffer and incubated at room temperature for 30 mins. The cells were washed with calcium indicator-free buffer to remove any dye that is nonspecifically associated with the cell surface, and then incubated for additional 30 mins to allow complete de-esterification of intracellular AM esters. Nicotine (100 µM) or KCl (50 mM) was then applied and the calcium influx measured. Memantine at 10 to 300 µM was added 15 mins before application of nicotine or KCl. Since consistent calcium influx induced by nicotine or KCl is obtained in the first 3 applications [16], memantine of different concentrations was applied in different preparations and one preparation was tested for one concentration of memantine. At the end of experiments, IC_50_ values of memantine on nicotine-induced calcium influxes with 95% confidence intervals [46] were calculated. Calcium fluorescence images were examined with an inverted fluorescence microscope (Leica, Wetzlar, Germany). Fluo-4 was excited at 488 nm, and emitted fluorescence was filtered with a 535±25 nm bandpass filter and read into a computer running MetaFluor software (Nihon Molecular device, Tokyo, Japan) and quantified.

### Drugs and Statistical Analysis

The following drugs were used: U46619, (-)-nicotine, N^ω^-nitro-L-arginine (L-NNA), papaverine (PPV), (±)-isoproterenol (ISO), and memantine (all from Sigma-Aldrich, St. Louis, MO, USA); Lovastatin (Calbiochem, USA); Sodium nitroprusside (SNP) (Riedel-de haën, Seelze, Germany); All drugs were dissolved in deionized water and added directly into tissue baths. The drug concentrations were the final concentrations in the bath. The sigmoidal dose-response curve fitting was measured and analysed using GraphPad Prism (GraphPad Software). Data were expressed as mean±SEM and analyzed by ANOVA followed by Scheffe post-hoc analyses. The value of *p*<0.05 was accepted as significant.
